# Technoeconomic Modeling of Plant-Based Griffithsin Manufacturing

**DOI:** 10.3389/fbioe.2018.00102

**Published:** 2018-07-24

**Authors:** Aatif Alam, Linda Jiang, Gregory A. Kittleson, Kenneth D. Steadman, Somen Nandi, Joshua L. Fuqua, Kenneth E. Palmer, Daniel Tusé, Karen A. McDonald

**Affiliations:** ^1^Department of Chemical Engineering, University of California, Davis, Davis, CA, United States; ^2^Global HealthShare Initiative, University of California, Davis, Davis, CA, United States; ^3^Center for Predictive Medicine, University of Louisville, Louisville, KY, United States; ^4^Intrucept Biomedicine, LLC, Sacramento, CA, United States

**Keywords:** biomanufacturing, economics, Griffithsin, microbicide, antiviral, cost, modeling

## Abstract

Griffithsin is a marine algal lectin that exhibits broad-spectrum antiviral activity by binding oligomannose glycans on viral envelope glycoproteins, including those found in HIV-1, HSV-2, SARS, HCV and other enveloped viruses. An efficient, scalable and cost-effective manufacturing process for Griffithsin is essential for the adoption of this drug in human antiviral prophylaxis and therapy, particularly in cost-sensitive indications such as topical microbicides for HIV-1 prevention. The production of certain classes of recombinant biologics in plants can offer scalability, cost and environmental impact advantages over traditional biomanufacturing platforms. Previously, we showed the technical viability of producing recombinant Griffithsin in plants. In this study, we conducted a technoeconomic analysis (TEA) of plant-produced Griffithsin manufactured at commercial launch volumes for use in HIV microbicides. Data derived from multiple non-sequential manufacturing batches conducted at pilot scale and existing facility designs were used to build a technoeconomic model using SuperPro Designer^®^ modeling software. With an assumed commercial launch volume of 20 kg Griffithsin/year for 6.7 million doses of Griffithsin microbicide at 3 mg/dose, a transient vector expression yield of 0.52 g Griffithsin/kg leaf biomass, recovery efficiency of 70%, and purity of >99%, we calculated a manufacturing cost for the drug substance of $0.32/dose and estimated a bulk product cost of $0.38/dose assuming a 20% net fee for a contract manufacturing organization (CMO). This is the first report modeling the manufacturing economics of Griffithsin. The process analyzed is readily scalable and subject to efficiency improvements and could provide the needed market volumes of the lectin within an acceptable range of costs, even for cost-constrained products such as microbicides. The manufacturing process was also assessed for environmental, health and safety impact and found to have a highly favorable environmental output index with negligible risks to health and safety. The results of this study help validate the plant-based manufacturing platform and should assist in selecting preferred indications for Griffithsin as a novel drug.

## Introduction

Griffithsin is a high-mannose binding lectin found natively in the marine red alga *Griffithsia* (Mori et al., 2005). The protein is composed of 121 amino acids and its monomer has a mass of approximately 13 kDa. Griffithsin forms a homodimer with six binding pockets with high affinity for mannose, a common sugar found at the terminal end of oligosaccharides on the surface of many enveloped viruses. The protein is thought to inhibit the entry of enveloped viruses into host cells as well as viral maturation and transmission events by binding to oligosaccharides on the viral envelope surface. Native Griffithsin and its analogs are the most potent HIV-1 entry inhibitors yet described, with EC50 values in the picomolar range (Mori et al., 2005; O'Keefe et al., 2009). Griffithsin also effectively inhibits transmission of HSV-2 (Nixon et al., 2013), HCV (Meuleman et al., 2011), SARS-CoV (O'Keefe et al., 2010), Ebola (Barton et al., 2014), and possibly other viruses yet to be studied. Importantly, Griffithsin appears devoid of cellular toxicity that is associated with other lectins. O'Keefe et al. conducted studies with explants of macaque and rabbit vaginal tissues *ex vivo* and showed that Griffithsin did not induce changes in the levels of cytokines or chemokines, nor did it alter lymphocyte levels in human cervical tissue nor elicit inflammatory responses in rabbit tissue (O'Keefe et al., 2009). The combination of extremely wide viral target range and demonstrated preclinical safety makes Griffithsin potentially useful as a prophylactic and/or therapeutic in multiple and diverse antiviral indications.

The potential indications for Griffithsin as a human prophylactic or therapeutic include its use as an active pharmaceutical ingredient (API) in vaginal and rectal microbicides. In spite of the value shown by pre-exposure prophylaxis (PrEP) drugs to prevent HIV transmission, issues of cost, side effects, the potential for development of viral resistance through chronic use of antiretrovirals (ARV) as prevention modalities, and access to PrEP drugs by under-resourced populations remain. These unmet needs could be met by the availability of affordable, safe and effective “on demand” antivirals, especially with Griffithsin as the API and its potential to control co-transmitted viruses such as HIV-1, HSV-2 and HCV during intercourse. Adoption of Griffithsin as a new biologic drug, especially in cost-constrained products such as microbicides, is predicated on the feasibility of a scalable manufacturing process that can supply market-relevant volumes of the API at an acceptable cost of goods sold (COGS).

Previously, we showed that recombinant Griffithsin can be expressed and isolated with high efficiency using transient gene expression in green plants (Fuqua et al., 2015a,b). Although the process described can be further optimized, the achieved pilot-scale expression yields of >0.5 g Griffithsin per kg of fresh (hydrated) green biomass (“fresh weight”; FW), recovery efficiencies of 60–90% overall, and Griffithsin purity of >99% of total soluble protein (TSP) are already impressive. In this study, we developed a technoeconomic model for Griffithsin manufacturing using a plant-based system with the goal of estimating API manufacturing cost and determined the factors that have the greatest impact on COGS. The output of our study should serve as a basis for additional process improvements, selection of a commercial-scale manufacturer, and should assist in the identification of future product targets for cost-sensitive markets such as prophylactic microbicides as well as those for less cost-constrained therapeutic indications.

Technoeconomic modeling was performed with the widely used SuperPro Designer modeling software (Intelligen, Inc., Scotch Plains, NJ, USA). The main analysis in this study was conducted using data available from pilot-scale manufacturing of Griffithsin in *Nicotiana benthamiana* plants using tobacco mosaic virus (TMV)-induced transient gene expression, and assuming that manufacturing would take place in an existing and fully equipped state-of-the-art plant-based biomanufacturing facility. Modeling costs based on existing resources of a contract manufacturing organization (CMO) instead of a “greenfield” build of a new facility was seen as the most likely scenario for launch of a new product. Our reasoning was that dedicated infrastructure could be built subsequently depending on market demand for the drug. As a result, we did not estimate capital equipment or total capital investment costs, and neglected depreciation, insurance, local taxes and factory expenses in the manufacturing operating cost analysis as these investments would have been made by the CMO. Our analysis assumed a 20% net profit margin/fee (Sood et al., 2017) assessed by the CMO and this figure was added to the production cost of the product to arrive at the final total product cost.

In addition to the technoeconomic analysis, an Environmental Health and Safety Assessment (EHSA) of the designed process was conducted using the method described by Biwer and Heinzle (2004) to evaluate the environmental, health and safety impact of Griffithsin manufacturing using the plant-based system, with the goal of assessing the sustainability of the process.

## Materials and methods

### Modeling software

The technoeconomic modeling for this study was performed using SuperPro Designer (“SuperPro”), Version 9.5 (Intelligen, Inc., Scotch Plains, NJ; http://www.intelligen.com/), a software tool for process simulation and flowsheet development that performs mass and energy balances, equipment sizing, batch scheduling/debottlenecking, capital investment and operating cost analysis, and profitability analysis. This software has been used to estimate cost of goods in a variety of process industries including pharmaceuticals produced by fermentation (Ernst et al., 1997) and plant-made pharmaceuticals (Evangelista et al., 1998; Zapalac and McDonald, 2007; Tusé et al., 2014; Nandi et al., 2016). It is particularly useful at the early, conceptual plant design stage where detailed engineering designs are not available or warranted. SuperPro was chosen because it has built-in process models and an equipment cost database for typical unit operations used in the biotechnology industry, such as bioreactors, tangential flow ultrafiltration and diafiltration, chromatography, grinding or homogenization, and centrifugation. There are some specific unit operations and processes used in this study that are currently not included in SuperPro, such as indoor plant cultivation, transplantation, plant harvesting and screw press/disintegrator. Such unit operations were addressed through the “Generic Box” feature of the application. Unless otherwise noted, the maintenance costs of major equipment, unit operation-specific labor requirements and costs (e.g., operators, supervisors), pure components, stock mixtures, heat transfer agents, power and consumables (e.g., filter membranes, chromatography resins) used in the analysis were determined using the SuperPro built-in equipment cost model and default databanks. Additional case study specific design parameters were selected based on experimental data from journal articles, patent literature, the authors' laboratories, interviews with scientists and technologists conducting the work cited, technical specification sheets or correlations, heuristics, or assumptions commonly used in the biotechnology and/or agricultural industry.

### Modeling protocol

Process flow and unit operations were derived from published methods and unpublished results obtained by the authors and collaborators who have participated in the development and scale-up of the process described and in the development of Griffithsin products. On the basis of this information, the SuperPro software was used to select and size equipment for each of the unit operations to achieve the desired production target (20 kg of purified Griffithsin/year), simulate the operations by performing material and energy balances, and specify and schedule all operations taking place within each piece of equipment to calculate material inputs and outputs and process times. Costs for raw materials, utilities, consumables, labor, laboratory QA/QC, waste disposal and equipment maintenance were then used to determine annual operating costs, and per-unit mass or per-dose costs ($/kg or $/dose).

The main case study model was based on an existing plant-based manufacturing facility, operating in batch mode, and excluded new capital investments and other facility dependent costs, except for equipment maintenance costs, which were included. For the downstream portion of the Griffithsin manufacturing process, an annual available operating time of 7,920 h (330 days, 24-h operation, or 90% available operating time per year) for the facility was used with indoor-grown *Nicotiana benthamiana* plants. Operating time was based on Holtz et al. (2015) for a similar facility, which was designed with overlapping utility capacity and in which the largest single utility unit can be down for maintenance and/or repairs and the utility loads can be maintained with redundant (spare) equipment. Likewise, per Nandi et al. (2016) it was assumed that the plants would be grown continuously throughout the year (8,760 h, or 365 days, 24-h operation, or 100% available operating time per year). Land costs, upfront R&D, upfront royalties, and regulatory/certification costs were neglected in the model as these costs can vary widely.

### Host plant species selection and justification

Griffithsin protein for this modeling study was produced in *Nicotiana benthamiana* host plants. This host is preferred for indoor protein manufacturing due to its metabolic versatility, permissiveness to the propagation of various viral replicons, and high expression yields achievable with a wide range of targets, as reviewed by Pogue et al. (2002), De Muynck et al. (2010), Thomas et al. (2011), Gleba et al. (2014), and others.

### Gene expression options

Griffithsin protein can be produced in plants in a number of ways. These include (a) stable expression in recombinant plants; (b) inducible expression in transgenic plants; (c) transient expression induced directly by tobacco mosaic virus (TMV) replicons; or (d) via agrobacterial vectors introduced into the plants via vacuum-assisted, or surfactant-assisted, infiltration (Gleba et al., 2014). Relative to stable transgenic plants, the advantages of speed of prototyping, manufacturing flexibility, and ease of indoor scale-up are clearly differentiating features of transient systems and explain why this approach has been widely adopted in the manufacture of many plant-made pharmaceuticals (Gleba et al., 2014). In our base-case analysis, we modeled expression of Griffithsin using TMV induction described in Fuqua et al. (2015b) and results from 3 pilot-scale manufacturing runs because these batches provided the most extensive and complete data set; however, this process has been corroborated in 6 additional manufacturing runs at pilot-scale or larger.

### Seed germination and plant growth

*Nicotiana benthamiana* host plants are generated from seed and propagated indoors under controlled environmental conditions until sufficient biomass is obtained for inoculation with the TMV vector carrying the Griffithsin gene. The process is summarized as follows. An *N. benthamiana* Master Seed Bank is generated from seeds obtained from the U.S. Department of Agriculture (USDA) Repository. For biomanufacturing, seeds from the TW-16 line are obtained in bulk and stored securely. The Master Seed Bank is qualified for germination rate (>95%), freedom from disease, and genetic uniformity, and stored in sealed containers under temperature-controlled conditions (5 ± 3°C). If the seed batch passes release tests, it becomes the Production Seed Batch and is used in the designated production run (“Working Seed Lot”). Seedlings are allowed to grow for 21 days under controlled environmental conditions (27 ± 2°C and 50% RH per Holtz et al., 2015). At this stage, the seedlings are transplanted to accommodate their larger size and moved to another growth room to await inoculation, as described in the following sections.

### Expression vector

The expression vector is constructed as described in O'Keefe et al. (2009). Briefly, a synthetic cDNA (GenBank no. FJ594069) encoding the 121-amino acid Griffithsin amino acid sequence is cloned into a TMV-based expression vector. In post-translational processing *in planta*, Griffithsin's amino-terminal methionine is cleaved and the N-terminal serine is acetylated. The construct containing the TMV vector backbone and Griffithsin gene insert are built into a plasmid that is propagated in the *E. coli* host strain DH10B (Fuqua et al., 2015b) and constitutes a Master Plasmid Bank. The Master Plasmid Bank is maintained in stocks at −20°C and is checked periodically for stability and insert fidelity. Excision via T7 polymerase produces free TMV transcript, which constitutes a Working Transcript Batch used to inoculate *N. benthamiana* plants 24 days after sowing and generate a TMV Virion Inoculum Batch 7 days post infection, which is checked for conformance to quality control criteria (e.g., infectivity, message fidelity, bioburden, stability) per Fuqua et al. (2015a). The TMV inoculum is then applied to host plants to initiate expression.

### Inoculation

The TMV inoculant is applied to the 24-day-old plant host production batch by high-pressure spray with an abrasive (diatomaceous earth), to introduce the virus into plant tissue. Once the TMV vector gains access to plant tissue, the virion decapsidates and the genomic RNA encodes for a polymerase/replicase to multiply the message. As described in Shivprasad et al. (1999) and Pogue et al. (2010), subgenomic promoters also drive expression of a movement protein (MP) to translocate the transcript throughout the plant, and a coat protein (CP) that encapsidates the RNA and reconstructs the virion that then self-propagates throughout the plant. Simultaneously, a subgenomic promoter (TMV U1) also drives expression of the Griffithsin gene, which is translated into Griffithsin protein. Plants at this stage are therefore induced to synthesize the API. Using this expression method, Griffithsin concentration *in planta* reaches a maximum without further increase typically 14 days post inoculation (optimized internally based on the amount of inoculum used). At this stage, the plants are ready for API extraction.

### Extraction of API

The API extraction procedure modeled is per Holtz et al. (2015) except that a 1:1 ratio of biomass:buffer is used. Briefly, the aerial parts of the plants (i.e., leaves, stems) containing accumulated Griffithsin are mechanically inverted and cut with a mechanical cutter. The harvested biomass is collected in baskets for transport to the extraction suite, to initiate downstream processing. The harvested biomass fresh weight (FW) is determined to calculate the volume of extraction buffer to be added, typically at a rate of 1 kg biomass FW:1 L buffer mix (100 mM sodium acetate, 300 mM sodium chloride, 20 mM ascorbic acid, 10 mM sodium metabisulfite). The pH is adjusted to 4.0 and the mixture is heated to 55°C for 15 min to help precipitate major host plant proteins. The heated mixture is passively cooled and filtered (0.3 μm cellulose filter) to yield a crude extract. The crude extract is stirred overnight at 4°C in the presence of bentonite and MgCl_2_. This procedure helps remove TMV coat protein (CP), which at this step represents the largest protein impurity in the extract. The suspension is filtered (0.3 μm cellulose filter) to remove aggregated TMV CP, yielding a clarified and partially purified API-containing solution and is then sterile-filtered (0.2 μm polyethersulfone filter). In-process controls are applied throughout downstream processing unit operations to determine reagent volumes and assess yield and quality at key steps.

### Purification of griffithsin

The partially purified extract is subjected to Capto^®^ MMC multi-modal chromatography (GE HealthCare) per Fuqua et al. (2015b) using a 2-step PBS gradient (90% and 100% phosphate buffered saline [137 mM NaCl, 2.7 mM KCl, 10 mM NaH_2_PO_4_, 2 mM KH_2_PO_4_]) at pH 7.4. The purified product consists of the drug solution in PBS, which is considered the Drug Substance (DS). The DS is release-tested per specification and is typically >99% pure (Fuqua et al., 2015b). The DS solution is typically bulk-packaged in inert bottles with screw cap closures per USP Class VI guidelines. Because container options vary, the final packaging step was not included in the model.

### Expression yield and recovery rates

Results used for modeling purposes were averages from 3 non-sequential manufacturing runs at pilot scale conducted at Kentucky BioProcessing LLC (“KBP,” Owensboro, KY, USA), the first of which was described in Fuqua et al. (2015b). These results have been corroborated by 6 additional production runs since. Under the conditions described, 0.52 g Griffithsin is expressed per kg plant biomass FW. Overall recovery efficiency by the method described is typically ≥70%, or ≥0.37 g Griffithsin/kg FW biomass.

### Manufacturing facility

To adequately meet the projected initial annual market demand for a rectal microbicidal formulation in the United States, approximately 6.67 million doses of Griffithsin API at 3 mg/dose would be needed. This translates into a production rate of 20 kg of purified Griffithsin API per year. The manufacturing facility to produce the required 20 kg of API per year was assumed to segregate production operations into two broad categories; namely, upstream production and downstream recovery and purification. To accommodate a large number of plants, the facility uses a vertical (layered) cultivation design with integrated irrigation and runoff collection system. Each rack is compatible with an integrated transportation infrastructure to move each tray to the next phase of the growth cycle.

The upstream portion of the facility houses unit operations for *N. benthamiana* propagation, inoculation with TMV vector, and Griffithsin protein expression and accumulation. These processes begin with seeding and end when the biomass is taken to harvest. The downstream portion of the facility begins at harvest and continues through purification of the Griffithsin DS. Upstream processing is assumed compliant with good agricultural practices (GAP), whereas downstream processing is subject to FDA current good manufacturing practice (cGMP).

The general layout of the upstream growth rooms was adapted from Holtz et al. (2015), and includes one germination chamber for seeds, one pre-inoculation room for biomass growth, and an isolated post-inoculation chamber where *N. benthamiana* inoculated with TMV expresses and accumulates Griffithsin. All plant growth was modeled to occur indoors using a vertical rack system with hydroponic irrigation. Plants are arrayed in equally sized trays under light-emitting diode (LED) light systems tuned to the optimized photosynthetic absorbance spectrum of *N. benthamiana* (composite blue/red spectrum: 25% 450 ± 10 nm wavelength/75% 660 ± 10 nm wavelength; Holtz et al., 2015) and are continuously illuminated.

The plants are rooted in rock wool cubes held in the trays by polystyrene foam floats and perfused with a nutrient solution (the components of which are listed in Supplementary Table 2 in Supplementary Material). Hydroponic irrigation is on a 12-h cycle and is accomplished via nutrient film technique (Holtz et al., 2015). We modeled a hydroponic system because the nutrient solution is recycled; hence, water is conserved, and fertilizer runoff is reduced although not eliminated. The mass of nutrient solution taken up by the plants, the cost of the nutrient solution per liter, and the mass of residual nutrient solution that goes to the wastewater treatment system are shown in Supplementary Table 1 in Supplementary Material. To ensure consistency of the nutrient solution, all water was assumed to be treated by reverse osmosis (RO) with solution-monitoring for proper pH and dissolved solids content.

### Environmental health and safety

A semi-quantitative environmental health and safety assessment was conducted by determining the hazardousness and mass of input materials used in the described upstream and downstream manufacturing operations as well as the hazardousness and mass of waste products generated. The method is referred to as “semi-quantitative” because the amounts of input and output components are quantified from SuperPro, but the “hazardousness” of each component is determined from the properties of the component (e.g., thermophysical properties, Material Safety Data Sheets, National Fire Protection Association (NFPA) ratings, etc.) per three qualitative classifications followed by assignment of a numerical value based on the classification (see Biwer and Heinzle, 2004).

### Process operations, materials and scheduling

The three phases of plant growth (i.e., seed germination, seedling growth and development pre-inoculation, and post-inoculation maturation) require a total batch time of 38 days in the upstream portion of the facility. Due to the protracted and continuous nature of plant cultivation, the upstream portion of the facility contains multiple concurrent batches staggered at different stages of growth. When one batch graduates to the next step of production (every 3.44 days), the trays containing the batch's biomass are cycled out and the corresponding rack space is immediately filled with a new rotation of trays. We divided the 38-day growth period into 11 concurrent batch periods, with one batch ready to enter downstream purification every 3.44 days. Table 1 is a summary of the number of plants, trays and batches that comprise the upstream facility at any given moment.

**Table 1:** Plant inventory in upstream facility.

	**Germination**	**Pre-Inoculation**	**Post-Inoculation**	**Upstream Total**
Number of plants	86,700	14,450	57,800	158,950
Number of batches	6	1	4	11
Batch residence time	21 days	3 days	14 days	38 days

For model building, batch schedules were calculated under the initial assumption of 24/7 operation for 330 days per year. Plant uptake of nutrients and growth were assumed to be linear reaching 15 g FW per plant at viral inoculation and then increasing in mass to reach 40 g FW per plant at harvest. A 5% failure rate of TMV inoculation was assumed (Pogue et al., 2002). The Griffithsin expression rate was fixed at 0.52 g/kg FW of harvested biomass, with a downstream recovery of 70%, based on pilot-scale results. Additionally, nutrient solution demand was assumed to match observed biomass growth rates assuming that for each kilogram of nutrient solutions, 0.5 kilogram goes into biomass and the remainder is considered aqueous waste.

The materials used, quantities and source are summarized in Supplementary Table 1 in Supplementary Material, together with clarifying comments and references that were used to assist in the calculations. Using the inputs shown in Supplementary Table 1, the upstream and downstream processes were modeled in SuperPro. The SuperPro files for this study can be downloaded from the following website: http://mcdonald-nandi.ech.ucdavis.edu/research/technoeconomic-analysis/.

## Results

### Upstream operations

**Figure 1:**
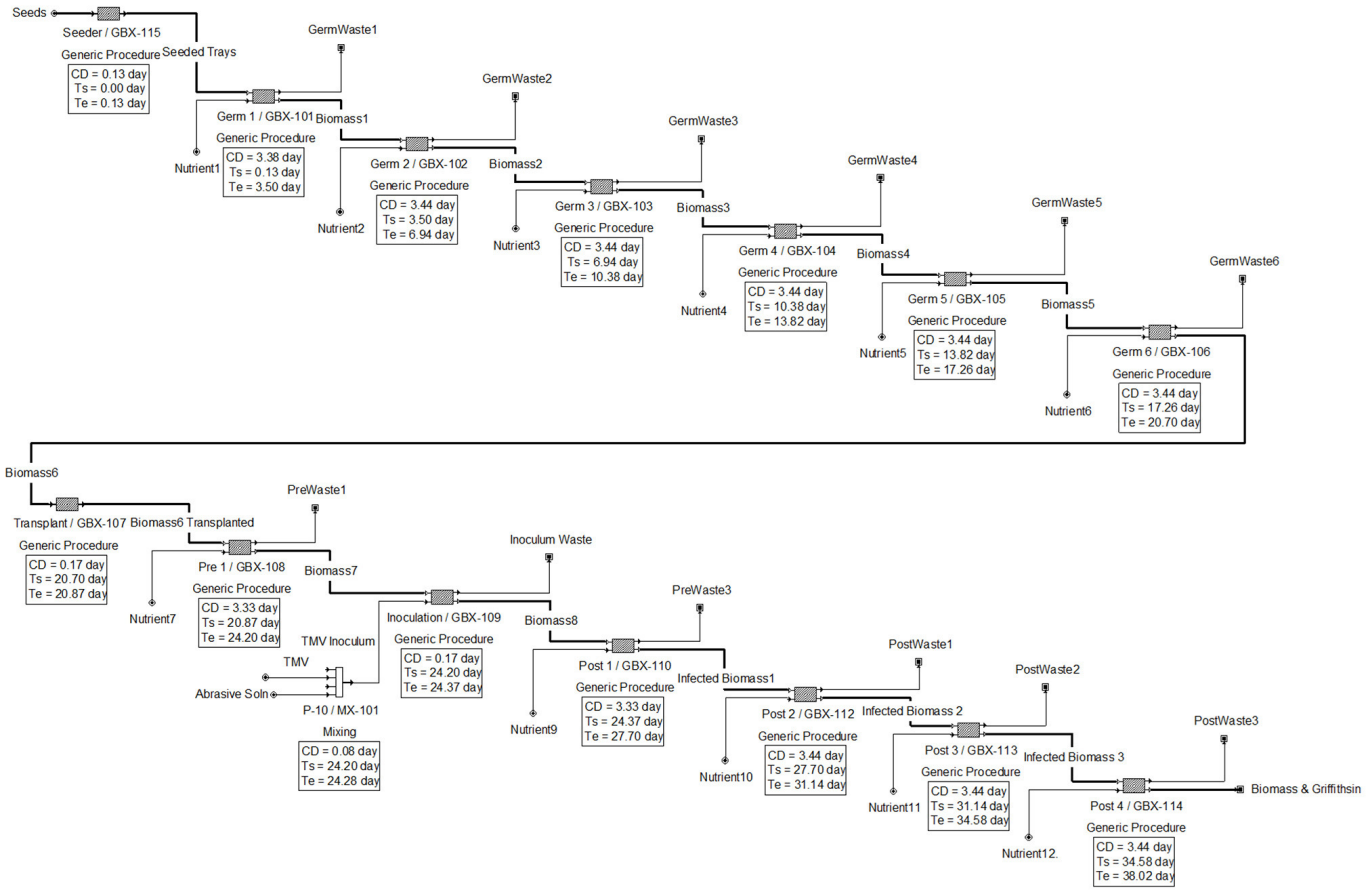
Upstream flow diagram for plant cultivation and Griffithsin production.

**Figure 2:**
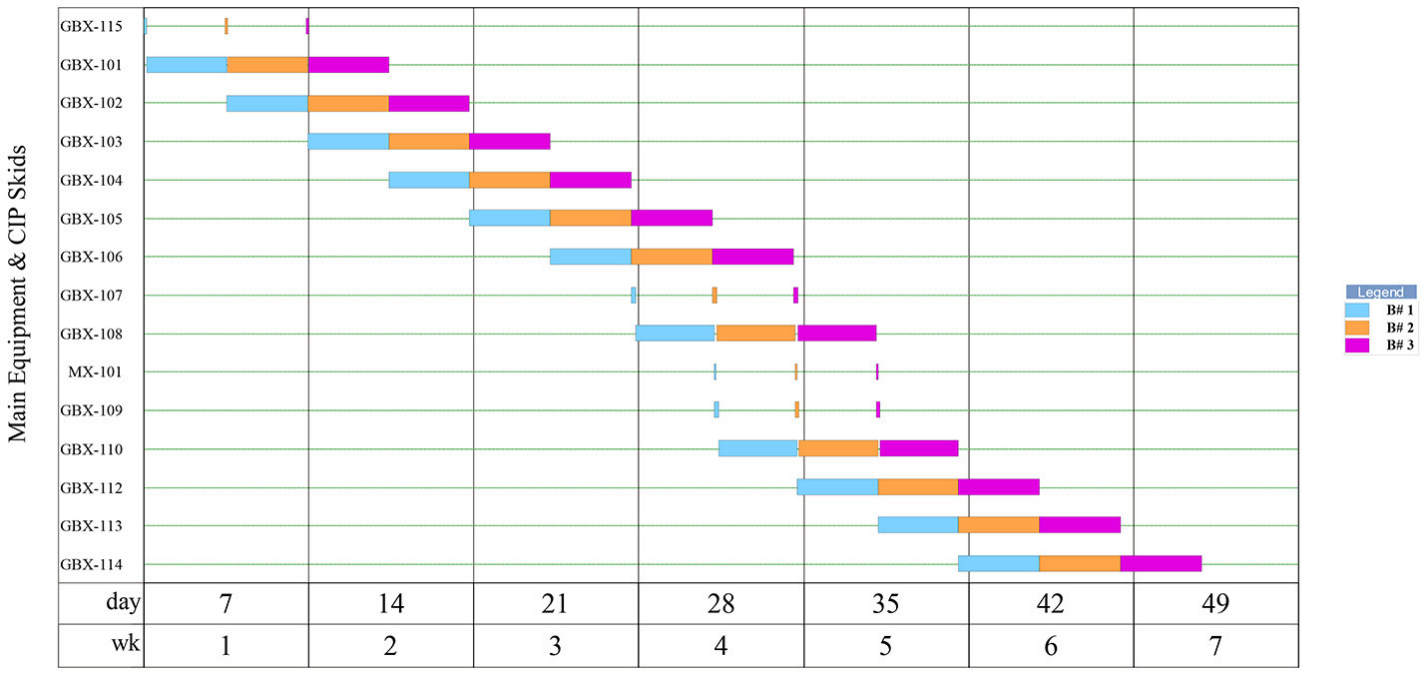
Equipment occupancy in upstream operations for 3 batches of Griffithsin production.

The results generated by the software for the upstream operations are shown in Figure 1, with scheduling shown in the equipment occupancy chart in Figure 2. The following descriptions elaborate on the schema presented in each figure.

Griffithsin recovery and purification was modeled as a batch process in a facility with an available operating time of 330 days a year for 24 h a day and 7 days a week. In each year, there are 95 batches total to produce 20 kg of purified Griffithsin API. Since the recovery and purification process only takes 1.6 days, the downstream facility has a significant down time of 2.78 days between batches. Overall, each batch requires 39.6 days from seed planting to formulating the final product, with 38 days upstream and 1.6 days downstream.

In Figure 1, the upstream processes are dictated by 11 concurrent batches (represented by generic boxes) with each batch being 3.44 days apart from each other. A batch basis of 3.44 days was chosen to decrease equipment idle time and thereby increase downstream equipment utilization efficiency. Despite the 39.6-day batch period and a 332-day operating year, in the model the batch time upstream was reduced to approximately 38 days and the operating year was increased to 365 days to reach the desired 95 batches per year. This was done because SuperPro reproduces uniform results for each year.

The goal of the upstream process operations is to produce sufficient biomass to enable isolation of 20 kg Griffithsin *per annum*. The modeling results show that each batch would produce 578 kg of biomass containing 300 g of Griffithsin, assuming an expression yield 0.52 g API/kg FW biomass (Fuqua et al., 2015b). Because induction was modeled using infection with recombinant TMV vector, the three main phases in upstream are germination, pre-inoculation, and post-inoculation. The duration of the phases in the model are 21 days, 3 days, and 14 days, respectively.

Each batch of *N. benthamiana* plants goes through a germination phase of 21 days and the germination room is designed with a capacity to grow the 86,700 plants necessary to reach the production goal. This step of the process uses 90 germination trays, each holding about 960 plants, distributed among 6 batches in the germination room.

After 21 days post germination, the *N. benthamiana* plants are transplanted to a lower density to enable further growth. Thus, seedlings from one germination tray are transplanted into three grow trays (with 320 plants per grow tray), meaning that there are three times the number of trays in pre- and post-inoculation, individually, than in germination. The plant density is 646 plants per m^2^ in the germination trays and 215 plants per m^2^ after transplantation. In practice, during transplantation each plant will spend only a few minutes away from its growth environment to minimize transplant shock and undue stress. In the model, the overall time was overestimated to be 3 h to accommodate other necessary procedures, such as moving the plants back to the tray stacks. The transplanted trays are relocated to pre-inoculation rooms that are designed to accommodate the increased area from transplanting for ~3 days. The pre-inoculation room contains 1 batch, each containing 45 trays with 320 plants per tray.

Recombinant TMV for inoculation is produced in and isolated from *N. benthamiana*. The plant growth model is the same as the rest of *N. benthamiana* plants. By using infected plants and the purification model defined by Leberman (1966), 4 mg of pure TMV per gram of infected plant material can be recovered (Leberman, 1966; Bruckman et al., 2014). Each batch is equivalent to 14,450 plants distributed on 45 trays. Less than 1 microgram of TMV virion is needed to inoculate each plant (Pogue et al., 2002). Thus, approximately 14.5 mg of TMV is needed per batch and the necessary amount of TMV to inoculate a batch can be produced from a single *N. benthamiana* plant. Multiple batches of TMV solution can be made simultaneously and stored at −20°C (Fuqua et al., 2015b). TMV production can be done at lab scale and equipment, labor and material costs are negligible (~$1,000) compared to the overall cost of plant maintenance.

The isolated TMV is incorporated in diatomaceous earth buffer solution at a concentration of 10 micrograms per 2.5 mL of diatomaceous earth buffer solution, which contains 1% by volume diatomaceous earth and 2% by volume of sodium/potassium-based buffer (Pogue et al., 2002). The selected inoculation volume of 2.5 mL is a safe middle value from the range suggested in the literature (e.g., 2-3 mL, Pogue et al., 2002). In the model, the estimated mixing and transfer time for the solution is 1 h, which starts at the beginning of post-inoculation, so the plants and solution enter the same stage together.

A forklift is used to transport the plants into the inoculation room. The plants are inoculated with the diatomaceous earth buffer solution described above with a high velocity spray. Inoculation machines are often custom made and consist of a conveyor traveling through an enclosed cylinder equipped with high pressure spray nozzles aimed at the plants' aerial structures. Once the inoculation is complete, the trays are conveyed to the post-inoculation growth room, which is similar in design to the pre-inoculation growth room; the main difference being its size. The post-inoculation room contains 4 batches at any given time for a total of 180 trays with 320 plants per tray.

The scheduling of 3 batches is summarized in the equipment occupancy chart in Figure 2. As shown, seeding, germination, transplant, pre-inoculation, inoculation, and post-inoculation occur sequentially, and the batches are staggered by 3.44 days.

### Downstream operations

**Figure 3:**
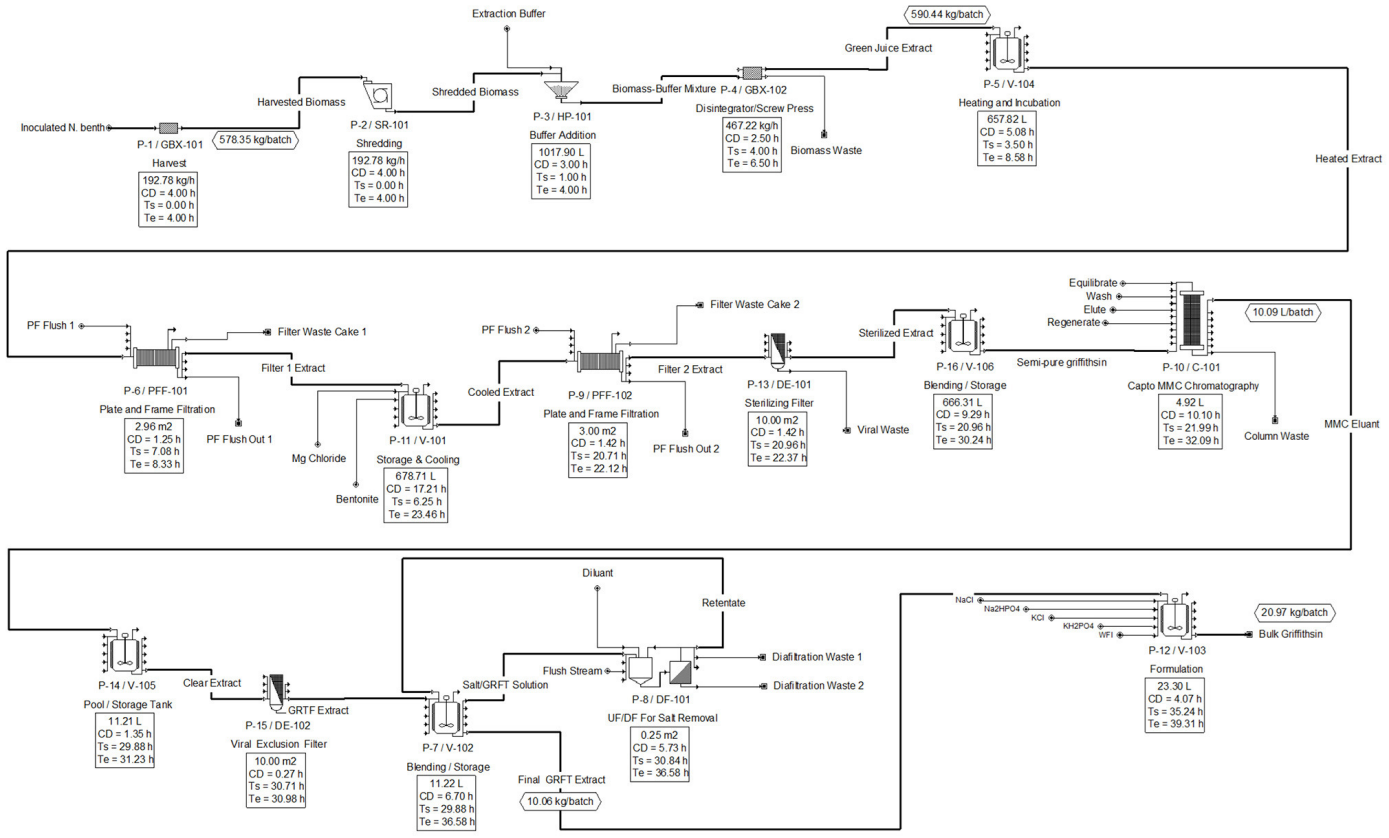
Downstream flow diagram for Griffithsin purification and recovery.

**Figure 4:**
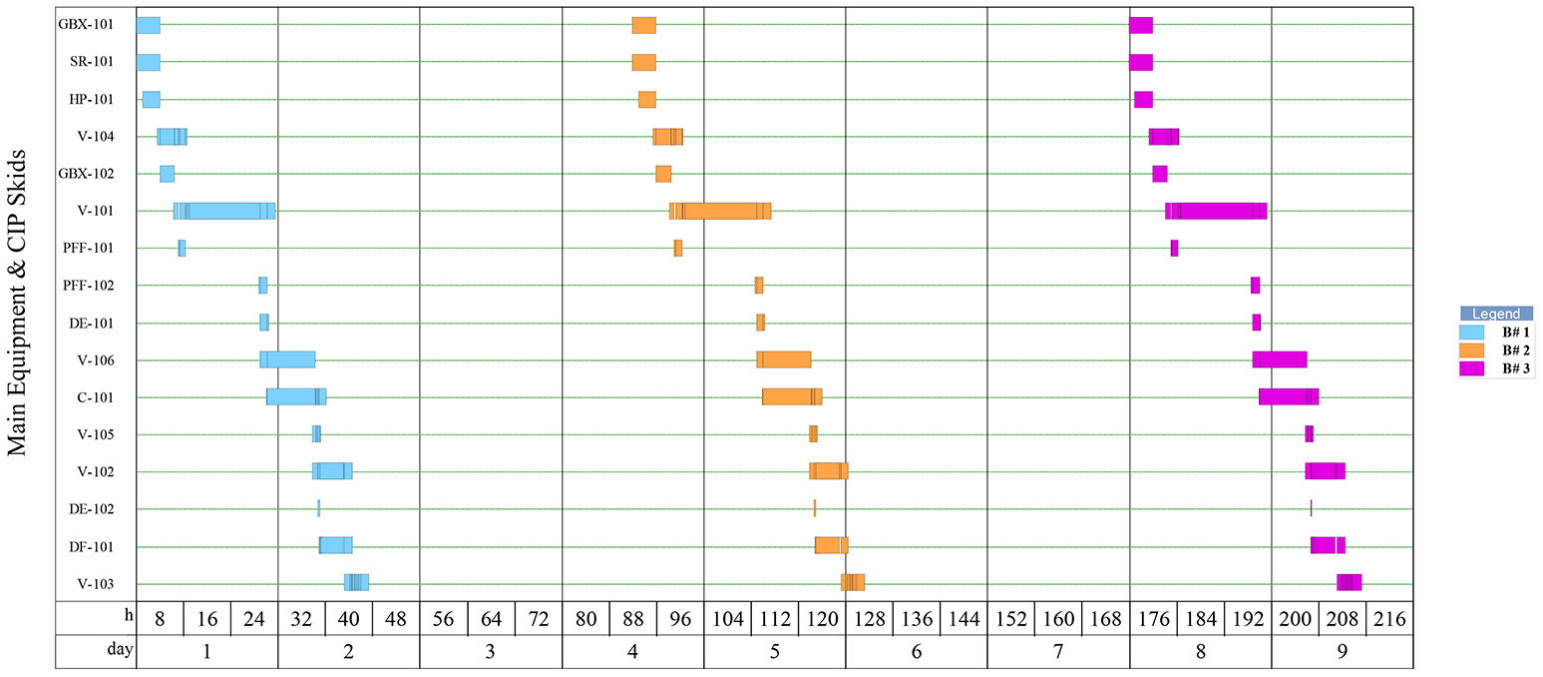
Equipment occupancy in downstream operations for 3 batches of Griffithsin purification.

The downstream unit operations developed in SuperPro are shown in Figure 3, with scheduling summarized in the equipment occupancy chart shown in Figure 4. The following descriptions elaborate on the schema presented in each figure.

At the end of each 3.44 day growing rotation cycle upstream, one batch of *N. benthamiana* plants is ready to be transferred to downstream processing. This is done by placing each tray of plants onto a conveyor system which leads them to the first phase of downstream operations. The matured plants are first harvested for the green biomass from which the majority (88%) of Griffithsin can be recovered with a single extraction. Additional Griffithsin could be recovered from fibrous material by reprocessing (O'Keefe et al., 2009) and from roots (which are not harvested); however, reprocessing was not included in this model. The automated harvester processes the 578 kilograms of biomass at a rate of 193 kilograms of biomass per hour. With an operational buffer time of 1 h, this process is thus expected to take 4 h.

As the biomass is processed by the harvester, it is directly fed into a shredder which further comminutes the biomass to improve Griffithsin recovery. The shredder operates at a capacity of 193 kg of harvested biomass per hour for 2.8 h. The shredded biomass is then mixed with an extraction buffer in a buffer addition tank. For every kilogram of plant material, 1 L of extraction buffer is added. Thus, for 578 kg of *N. benthamiana* in a batch, approximately 578 L of extraction buffer are added.

The resultant solid-liquid mixture has a total volume of about 1,135 L and is sent through a screw press, which is represented as a generic box in the model. The screw press separates the solid-liquid slurry leaving a main process fluid stream of plant extract and a waste stream of biomass. The extract solution contains Griffithsin as well as the host and viral protein impurities. A loss about 12% of the original starting Griffithsin was modeled assuming it to be non-liberated from the homogenized biomass. The removal of the biomass leaves a main process stream that contains about 585 L (590 kg/batch).

To facilitate the aggregation of proteinaceous impurities, the extract solution is transferred into a mixing tank and heated to 55°C for 15 min. The mixture is passively cooled and simultaneously transferred out of the tank and fed into the first 0.3 μm plate-and-frame filter. The extract solution is filter-pressed at 25–30 psig to remove the aggregated protein impurities. Filtering has a process time of 1 h and requires a filter area of 3 m^2^ to handle the 590 kg/batch of the process stream. At this stage, the process loses a further 8% of the Griffithsin but removes all the RuBisCO (ribulose-1,5-bisphosphate carboxylase/oxygenase) and 87% of the TMV coat protein impurities. The filtrate from this step is transferred to a second mixing and storage tank, mixed with bentonite clay and magnesium chloride, and stored at 4°C for a 12-h period. This stage is the bottleneck operation for the downstream process. After the 12-h incubation, the solution is filtered through a second 0.3 μm filter press and a 0.2 μm inline sterilizing filter. These operations remove the remaining protein impurities leaving a Griffithsin extract with greater than 99% purity but at the cost of losing 6% of the Griffithsin.

The second plate-and-frame filter has a filter area of about 3 m^2^ and will process all of the extract in 1 h. There is approximately 222 g of Griffithsin per batch at the end of the filtration phase. Following the filtrations steps, the Griffithsin extract solution is collected in a storage tank and further purified using an AxiChrom column with Capto MMC resin to remove residual color and potential non-proteinaceous impurities.

To accommodate the 222 g of Griffithsin in solution, 4.9 L of MMC bed resin is needed at a 45 mg/mL binding capacity (per product specification sheet). The order of the operations for this chromatography step are: Equilibrate, load, wash, elute, and regenerate. In total, chromatography requires 10 h with the load step taking the longest, at 8 h, because approximately 600 L of solution are processed. Chromatography is necessary to decolorize the extract at the expense of losing 4% of the Griffithsin, giving a remaining Griffithsin mass of 210 g per batch.

The 10 L of eluant process fluid is sent through a viral clearance filter and transferred into a pool/storage tank. Subsequently, the extract is sent through an ultrafiltration/diafiltration cycle to remove salts introduced in the chromatography column. After ultrafiltration, the product is transferred into a storage tank to be mixed with the final formulation components. The concentrated Griffithsin is diluted to give a concentration of 10 g/L Griffithsin in 10 mM Na_2_HPO_4_, 2.0 mM KH_2_PO_4_, 2.7 mM KCl and 137 mM NaCl at pH 7.4. The final volume of the DS is 21 L per batch.

As shown by Figure 4, each batch in the downstream requires 39 h of process time which includes all SIP and CIP operations. As batches move from the upstream portion of the facility every 3.44 days, the remaining time left over in the downstream is set as slack time in the model that may be dedicated toward repair, maintenance, etc.

### Economic calculations

**Figure 5:**
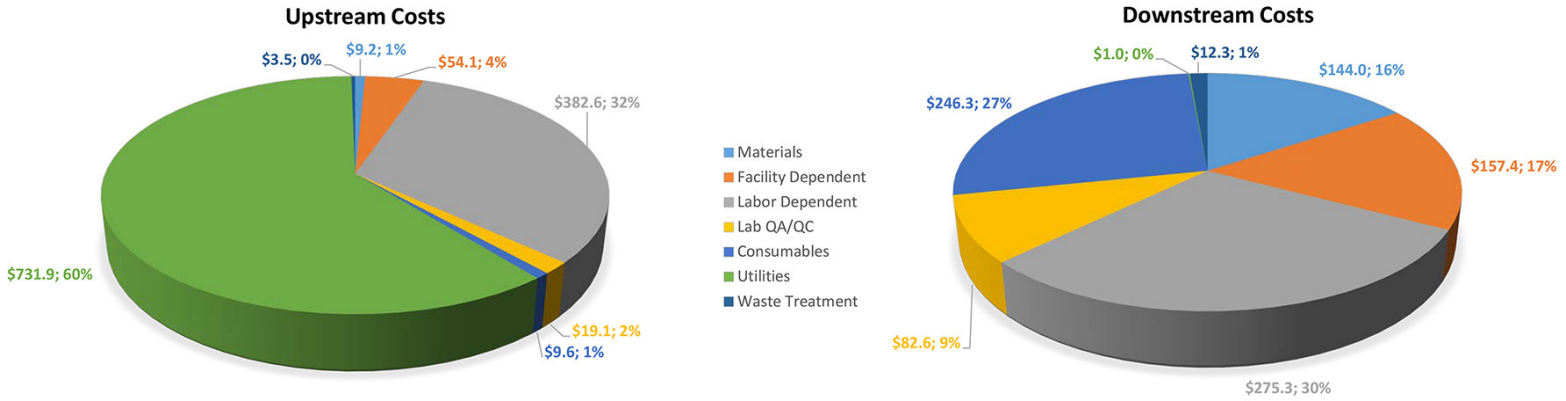
Upstream and downstream cost contributions by process category (units in $000).

The assumptions and results developed in SuperPro were used to calculate the economics of the process described. Table 2 shows the total operating costs segregated individually for upstream and downstream components. Figure 5 displays process category cost contributions graphically, including percentages of total costs. In upstream operations, the largest cost components are utilities ($731,857) and labor ($382,567), representing 61% and 32% of total upstream costs, respectively. In downstream operations, labor-dependent costs ($275,286) are the highest contributors at 30% of total downstream costs, followed by consumables ($246,325) at 27% of total downstream costs. Overall, the upstream component represents nearly 57% of the total Griffithsin production cost, which is calculated as just over $106/g protein. For a microbicide dose of 3 mg, the per-dose manufacturing cost is $0.32, excluding any CMO fee.

**Table 2:** Summary of upstream and downstream production costs.

**Process component**	**Upstream**	**Downstream**	**Total**
Materials (annual)	$9,200	$143,976	$153,176
Facility dependent costs (annual)	$54,050	$157,400	$211,450
Labor dependent costs (annual)	$382,567	$275,286	$657,853
Lab QA/QC (annual)	$19,128	$82,586	$101,714
Consumables (annual)	$9,597	$246,325	$255,922
Utilities (annual)	$731,857	$1,002	$732,859
Waste treatment (annual)	$3,540	$12,291	$15,831
Total operating expenses excluding depreciation ($/year)	$1,209,940	$918,866	$2,148,806
Total operating expenses excluding depreciation ($/batch)	$12,736	$9,672	$22,408
Unit production cost or COGS ($/gram of Griffithsin)	$60.51	$45.95	$106.46
(% of total)	(56.8%)	(43.2%)	(100%)
COGS of 3-mg dose of Griffithsin	$0.18	$0.14	$0.32

### Environmental health and safety

An environmental health and safety assessment was also conducted for this case study following the method of Biwer and Heinzle (2004) and the results are found in Supplementary Tables 2–4 in Supplementary Materials. Overall, the process uses chemicals that are not harmful to people or the environment, as can be seen by the low magnitude of input and output Environmental Factor values (typically less than 0.325 on a 0–1 scale) in Supplementary Table 4. The biggest causes for concern (based on the environmental indices) are TMV in the residual biomass, and sodium hydroxide and phosphoric acid used in clean-in-place operations, if released to the environment; however we included costs for a thermal or chemical deactivation step for the TMV-contaminated biomass and pH neutralization for the acid and base cleaning agents which would eliminate the environmental impact of these components. It should also be noted that the upstream nutrient compounds can be more efficiently recycled to increase nutrient utilization by the plants and reduce water/soil impact.

Waste compounds in the downstream process are disposed of through wastewater and biowaste treatment. An aggregate disposal cost of $0.01 per liter of non-TMV-contaminated aqueous streams and $0.1 per kg of biowaste is assigned in SuperPro for expenses related to wastewater disposal and thermal/chemical deactivation of biowaste streams. Compounds introduced during or after the post-inoculation step in the upstream facility are considered as biowaste since they may contain TMV. This includes spent nutrient solution in the post-inoculation step and retentate streams from plate-and-frame and dead-end sterilizing filtration skids. Disposal of TMV-contaminated materials poses low environmental risk. There is extensive industrial experience in disposing of TMV-contaminated materials, which can be rendered non-infective by treatment with bleach, heat or detergents, diluted and disposed of as municipal waste (Pogue et al., 2002).

## Discussion

### Conclusions based on modeled parameters

The facility modeled can annually produce 20 kg of the potent antiviral Griffithsin for use in microbicide products. The host used in our modeling was *Nicotiana benthamiana*. This species was selected because of its aforementioned productivity, but also because our previous report on technoeconomic modeling of *Nicotiana*-produced therapeutic and industrial products (Tusé et al., 2014; Nandi et al., 2016) prefaces the work reported herein. In addition, the use of *Nicotiana* for production of clinical trial materials is also familiar to FDA and other regulatory agencies, thus facilitating *Nicotiana's* acceptance in regulation-compliant manufacturing (Streatfield and Howard, 2003; McCormick et al., 2008; Bendandi et al., 2010; Tusé, 2011; Gleba et al., 2014).

The API is manufactured in the host *Nicotiana benthamiana* using tobacco mosaic virus (TMV) as the expression vector. The upstream plant growth and Griffithsin production operations are adapted from the facility layout detailed by Holtz et al. (2015). Over 158,000 plants are housed in vertically stacked hydroponic grow racks, fitted with high-efficiency LED lights. The environment is controlled and monitored for compliance with good agricultural practices (GAP). Each batch of 14,450 plants grows over the course of 38 days and yields a total of 578 kilograms (fresh weight) of biomass. Ninety-five batches are seeded and grown annually, with one batch reaching harvest every 3.44 days.

The downstream Griffithsin extraction and purification process is scaled up from the pilot industrial scale process presented by Fuqua et al. (2015b). An expression rate of 0.52 grams of Griffithsin per kilogram of biomass (fresh weight) and a downstream recovery of 70% were used in the base case and give a combined yield of 0.370 grams of Griffithsin per kilogram of harvested biomass. Sterile filtration and CIP/SIP systems facilitate compliance with cGMP guidelines. Downstream processing commences upon the completion of an upstream batch and takes 39.3 h. The stable final formulation is >99% Griffithsin as the API with negligible endotoxin levels.

In the model, the upstream costs account for nearly 57% of the total cost of Griffithsin production. Containing both upstream and downstream losses of the protein could significantly reduce COGS. Approximately 12% of the protein API is non-liberated from the homogenized biomass (reprocessing was not modeled) and 18% is lost during downstream polishing steps. Based on the data and assumptions employed in the current analysis, the unit production cost of Griffithsin is estimated to be $0.32 per dose (3 milligram).

The model was based on published designs for a commercial-scale facility and pilot-scale data on Griffithsin production adapted to the facility described. This type of modeling is useful for determining ranges of API selling price, production capacity and expression level requirements for commercial supply and profitability.

In this study we modeled the manufacturing of Griffithsin through a contract manufacturing organization instead of a greenfield build of a new facility because we assumed that that would be the most prudent approach to launching a new product. If the product manufactured using the process modeled is used directly as a vaginal rinse or rectal enema, the additional costs post manufacturing would include transportation, storage, insurance, distribution, marketing, etc., none of which were modeled in this manufacturer-level analysis. If the Drug Substance produced via the process analyzed is further formulated (e.g., as the API in gels, suppositories, or condom additives), or used as a component of another device (e.g., vaginal ring), those costs and other product-specific costs would be additive and were also excluded from our manufacturer-level analysis.

### Potential impact of cost on uptake of griffithsin-containing microbicides

The cost of goods calculated by the current model reflects the manufacturer's cost of production. We are less certain about the wholesale price of the drug because there is no standard “off-the-shelf” profit margin that can be added to toll manufacturing cost to arrive at a standardized answer. Often scale up to commercial launch volumes of a product requires additional process development and optimization, validation batches, etc., which lead to negotiated transfer prices depending on volume, duration of engagement, license fees, export duties, and other factors, all of which would impact the cost of bulk Griffithsin. Nevertheless, for this discussion we assumed a manufacturer's fee of 20% of COGS for a total production cost of bulk Griffithsin Drug Substance of $0.38/dose. Additive formulation, storage, distribution, insurance, marketing, sales margins and other costs could lead to a consumer-level use cost of $1-2/dose (i.e., ~3 to 5-times the production cost and <1 to 5 times the price of a male condom, which varies widely depending on material, features and quantities purchased).

This technoeconomic analysis emphasized Griffithsin's use in microbicides because such products arguably represent the most price-constrained applications of this new drug. We cannot define the target retail price of a Griffithsin microbicide; there is no market reference price for microbicides since no commercial microbicides yet exist. For perspective, the user cost of a Griffithsin microbicide can be benchmarked against pre-exposure prophylaxis (PrEP) with traditional male condoms and PrEP with microbicides containing antiretroviral (ARV) drugs as a newer alternative. Analyses have been conducted on the cost of prevention modalities and the cost savings to the healthcare system enabled by preventing HIV transmission, with prevention being far more cost effective than treatment in most scenarios (e.g., Pretorius et al., 2010). Walensky et al. (2012) conducted an analysis of the cost-effectiveness of a Tenofovir-based PrEP microbicide in South African women. In their cost modeling of a vaginal gel, they multiplied the product cost of $0.32/dose times 2 (product must be applied twice, pre- and post-intercourse) and by 7.2 (average sex acts per woman-month) to arrive at a product use cost of approximately $5/woman-month. However, the price of the microbicide gel used in the study was assumed and region-adjusted and hence pricing in other countries may be different. Terris-Prestholt et al. (2014) estimated Tenofovir gel prices of $0.25–0.33 per dose, provided that the gel was used in combination with a condom ($0.20–2.00 each; Planned Parenthood, 2018), from which an additive cost of use (single-use condom plus double-dose microbicide gel) of $7–$12/person-month can be derived. Assuming the same average use rate (7.2 applications/month) of a Griffithsin-containing microbicide applied singly without a condom and priced at $1.00–$2.00 per dose, the cost of use would be $7– <$15/person-month.

Whether a higher cost of use discourages adoption of Griffithsin-based microbicides by men and women remains to be shown. A market study by Darroch and Frost (1999) of the Alan Guttmacher Institute consisted of detailed interviews of a cross-section of 1,000 sexually active women aged 18–44 in the continental United States. Their statistically rigorous survey identified levels and predictors of women's concerns about STDs (including HIV transmission) and interest in microbicides, as well as their preferences regarding method characteristics and likelihood of usage versus price of product, with survey sample results extrapolated to the national level. The results showed that of the estimated 12.6 million women aged 18–44 interested in microbicides and concerned about STDs, including HIV, 11.5 million (91%) would still be interested in the method even if it were not 100% effective, and 11.0 million (87%) would remain interested even if the microbicide did not protect against STDs other than HIV. The same study found that women's predicted use of a microbicide was affected by price, but interest was still high at $2 per application, or roughly up to 5-times the average price of a male condom. The survey concluded that more than seven million sexually active women in the USA would be interested in a vaginal microbicide even if the product only protected against HIV, was only 70–80% effective and cost them $2 per application (Darroch and Frost, 1999). That conclusion was arrived at in 1999; the $2 per application cost back then would be $3.05 in 2018. One can conclude from these results that there is interest in effective yet inexpensive, self-administered HIV and STD prevention modalities even if such products might cost more than conventional prevention methods.

The Darroch and Frost analysis was conducted nearly 20 years ago, and the interviews were limited to women practicing vaginal intercourse. To our knowledge, a more recent study linking likelihood of product use and price sensitivity has not been conducted, or at least not reported, to include other populations of potential microbicide users such as heterosexual couples practicing anal sex or gay men practicing unprotected rectal intercourse. Nevertheless, the 1999 study established an initial price point and price sensitivity for potential users of microbicides in the USA.

Griffithsin has a broader spectrum of antiviral activity than HIV-specific PrEP agents, including activity against HSV-2 and HCV, which are co-transmitted with HIV-1 (Meuleman et al., 2011; Nixon et al., 2013). Hence, Griffithsin might command a higher price due to its broader antiviral activity and its potential to obviate prevention and treatment costs for co-transmitted viruses.

In the USA, the cost of the oral PrEP drug Truvada (emtricitabine and tenofovir disoproxil fumarate) ranges from $1,300 to over $1,700 per month (https://www.goodrx.com/truvada) for the uninsured, but treatment is typically covered by insurance with user co-payments of $80–$150 per month. So even if a Griffithsin-containing microbicide sold for $5 per application (e.g., $50 per 10-use pack), a user of 2 packs per month would pay $100 for the microbicide, which is in the range of PrEP, with the potential added benefit of controlling co-transmitted viruses.

Consumers in wealthier economies might be receptive to microbicides costing $1–2 or even more per dose; however, consumers in lesser-developed economies might find $1–2/dose to be prohibitive. Hence, absent subsidies, there exists a continuing need to lower COGS for APIs such as Griffithsin.

We can conclude that a COGS of <$0.40/dose of Griffithsin DS as determined in this study, and an estimated user cost of $1–2/dose, might enable at least some simpler formulations of the drug (e.g., rinses or enemas) to be economically marketed. For more complex formulations and delivery systems, or for higher doses of the drug, lower COGS for bulk Griffithsin would be desirable.

### Environmental impact of plant-based griffithsin manufacturing

The environmental assessment of the plant-based production of Griffithsin indicates low impact, particularly if the plant nutrient solutions are recycled in a hydroponic system and if waste streams containing TMV are treated in a biowaste heat or chemical treatment process. The assessment method used, although semi-quantitative, utilizes mass input and output stream data generated by SuperPro, along with independent assessment of compound toxicity and/or environmental impact (for example using Material Safety Data Sheet information), and allows comparison between alternative production strategies, process configurations or chemical components used in the manufacturing process.

Our low environmental impact assessment for plant-based manufacturing should compare favorably with fermentation-based approaches to producing Griffithsin (Giomarelli et al., 2006). In the latter, the complexities of purification suggest less efficient utilization of materials and higher disposal volumes, although a side-by-side environmental analysis between the two platforms was not conducted in this study.

### Modifications and improvements

Upstream, Griffithsin expression rates were based on empirical findings using TMV whole virion as the expression vector, which can achieve typically 0.5–1.0 g Griffithsin/kg plant biomass (Fuqua et al., 2015a). An average pilot-scale expression rate of 0.52 g/kg was used in our model (Fuqua et al., 2015b). Although this expression level is quite good for TMV, higher Griffithsin expression levels can be achieved with different technology. For example, Nomad Bioscience GmbH (Halle, Germany) has achieved Griffithsin expression in *N. benthamiana* exceeding 2.5 g Griffithsin/kg FW biomass using Nomadic™ agrobacterial vectors applied to plants either through vacuum infiltration or agrospray (Hahn et al., 2015), albeit these results were obtained in small-scale studies. The utilization of such an induction process instead of TMV virions could further improve process economics. For example, even with the same recovery efficiency of 70% assumed in the current model, the output of Griffithsin at the higher expression level would be 1.75 g API/kg plant material, instead of the current 0.37 g/kg; this represents more than 4.7-times the modeled output of protein per kg biomass. Under such conditions, the costliest parts of the current process, namely biomass production and upstream procedures, would be lowered by the reduced biomass needs to produce the required 20 kg/year of API. Although a full analysis of the cost of agrobacterial inoculation for Griffithsin production needs to be conducted, we know from similar analyses (e.g., Nandi et al., 2016) that economics can be favorably impacted by higher expression efficiencies. We can therefore envision that by using a more efficient induction process the per-dose production cost could be less than the current $0.32. Still other gene expression methods can be considered, including using transgenic plants expressing Griffithsin either in constitutive or inducible systems (Werner et al., 2011; Gleba et al., 2014), which could also lead to higher API accumulation in host plant biomass and potentially lower COGS (Tusé et al., 2014). Increasing expression yield upstream might shift costs to downstream operations to handle process streams with higher concentrations of API. Definition of the comparative cost benefits of these improvements relative to the current process modeled awaits a subsequent evaluation.

From a process standpoint, improvements in the efficiency of lighting technologies and/or incorporating solar panels would reduce upstream utilities costs, one of the major contributors to the upstream operating costs. Improving hydroponic nutrient utilization through recycling and minimizing runoff in the simulation model will reduce raw material costs as well as aqueous waste disposal costs, thereby reducing the COGS.

In the downstream portion of the process consumables play a major role, particularly dead-end filters and plate-and-frame filters; if these could be replaced with tangential flow filtration systems that utilize reusable, cleanable ceramic filters, downstream operating costs could be further reduced. At the time of this writing, such systems were being considered and their impact on Griffithsin COGS will be the subject of a future analysis.

## Author contributions

AA, LJ, GK, KS, SN, and KM contributed to the conception and design of the study. AA, LJ, GK, and KS conducted initial modeling calculations, provided information for Supplementary Tables 2–4, organized preliminary results and prepared an initial report on the findings. DT, JF, and KM provided additional data inputs and further refined the scope of the model. KM developed the final SuperPro model and TEA results. DT wrote initial and final drafts of the manuscript, including the cost-of-use analysis and consumer price sensitivity discussion, with primary editorial input from JF, KP and KM. KP provided critical reading of the manuscript. All authors contributed to manuscript revision, read and approved the submitted version.

### Conflict of interest statement

The authors declare that the research was conducted in the absence of any commercial or financial relationships that could be construed as a potential conflict of interest.
