# Supplementary material for: Technoeconomic Modeling of Plant-Based Griffithsin Manufacturing
Source: Front Bioeng Biotechnol. 2018 Jul 24;6:102. doi: 10.3389/fbioe.2018.00102 (PMC6066545; doi:10.3389/fbioe.2018.00102)
Supplement: Supplementary file 1 [file Data_Sheet_2.pdf]

Supplementary Material

Technoeconomic Modeling of Plant-Based Griffithsin Manufacturing

Aatif Alam, Linda Jiang, Gregory Kittleson, Kenneth Steadman, Somen Nandi, Joshua Fuqua, Kenneth Palmer, Daniel Tusé*, and Karen A. McDonald

*** Correspondence:** Daniel Tusé, Ph.D.: daniel.tuse@intrucept.com

1 Assumed Manufacturing Inputs

Supplementary Table 1 is a listing of materials used in the manufacturing model, their quantities and sources, together with clarifying comments and references that were used to assist in the calculations.

Supplementary Table 1. Summary of assumed inputs for manufacturing operations.

Item or Input	Quantity	Assumptions	Source
Griffithsin overall recovery	70%	Based on published results of pilot-scale production studies	Fuqua et al. 2015b
Batch time (duration)	39.6 days	Assumes a 24-day plant growth period, viral inoculation, 14-day post-inoculation incubation and 1.6-day downstream processing times	Fuqua et al. 2015b
Expression level	0.52 g/kg FW	0.52 g Griffithsin API per kg FW plant host biomass	Fuqua et al. 2015b
Batches per year	95	Based on downstream processing time (1.6 days) and 330 operating days per year. With 95 batches per year gives a downstream scheduling margin of 1.9 days.	Calculated
Batch biomass	578 kg/batch	Based on the design premise of 20 kg Griffithsin API/year production, recovery of 70% and expression level of 0.52 g/kg FW.	Calculated
Griffithsin production (prior to downstream processing)	301 g/batch	0.52 g/kg x 578 kg/batch	Calculated
Plants per batch	14,450	Batch biomass of 578 kg/batch divided by the weight of a plant at harvest (40 g)	Calculated
Plant inventory	158,950	Total plant occupancy in facility (6 germination batches, 1 post-transplantation batch, and 4 post-inoculation batches = 11 batches total at 14,450 plants per batch)	Calculated
Cost of plant nutrient solution	$0.06/L	Prepared on site using USP water	Nandi et al. 2016

Item or Input	Quantity	Assumptions	Source
Nutrient solution used	Days 1-24: 420 L per batch<br>Days 24-38: 720 L per batch	One kg of nutrient broth is converted to 0.5 kg biomass and 0.5 kg waste. FW of each plant at time of viral infection (day 24) is 15 grams and FW of each plant at day 38 is 40 grams.	Calculated
Mass of seed	0.0001 g	10,000 seeds per gram	Nandi et al. 2016
Cost of seed	$0.95/1,000 seeds	Produced in house	Nandi et al. 2016
TMV vector mass required per plant	1 microgram	1 microgram TMV virion per plant for inoculation	Pogue et al. 2002
TMV concentration	10 micrograms per 2.5 mL	10 microgram TMV virion mass in 2.5 mL of diatomaceous earth inoculant carrier	Calculated, and Pogue et al. 2002
Diatomaceous earth	1%	1% wt/vol of inoculant abrasive solution	US Patent 20060218667 A1
Buffer salts in inoculum	2%	Sodium/potassium phosphates assumed to be 2% of 0.5M phosphate buffer solution	US Patent 20060218667 A1
Diatomaceous earth cost	$55.60/kg		Calculated from vendor survey
Phosphate buffer cost	$44.80/kg		Calculated from vendor survey
Diatomaceous earth solution cost	$2.64/kg	Assumes USP water as carrier	Calculated
TMV infection rate	95%	95 plants infected per 100 plants inoculated with TMV suspension sprayed aerially at 100 psi pressure	Pogue et al. 2002
TMV purification yield	4.5 mg/kg	4.5 mg TMV recovered per gram FW biomass	Bruckman et al. 2014; Leberman et al. 1966
Griffithsin loss in downstream per step	12%<br>8%<br>6%<br>4%<br>30% total	Screw Press<br>First Plate and Frame Filtration<br>Second Plate and Frame Filtration<br>Chromatography	Fuqua et al. 2015b and calculated
Resin binding capacity	45 mg/mL	Manufacturer specification for CaptoMMC	GE Life Science
Chromatography column	25 cm diameter	Calculated from a bed height set at 10 cm and binding capacity of 45 mg/mL and 80% resin binding utilization	Calculated
Column linear velocity	150 cm/hr	Selected to provide a residence time of ~4 min	Calculated
Resin Replacement Frequency	40 cycles		GE Life Science
Resin Cost	$1,500/L		SuperPro Default

Item or Input	Quantity	Assumptions	Source
Upstream Operator Labor Hours Per Year	8,316 labor-hrs/year	Operation-specific operator labor-hour per process operation: 1 labor-hr per process hour for seeding, transplantation and inoculation operations, 0.06 labor-hours per plant growth operation during a 21 day germination period, 0.11 labor-hours per plant growth operation during a 17 day post-transplantation period<br>Model assumes 100% of labor hours devoted to process operations	Calculated
Upstream Labor Cost Per Year	$382,567/year	Based on Upstream Operator Basic Pay Rate of $20/hr and including 40% for benefits, 10% for operating supplies, 20% for supervision and 60% for administration = $46/hour adjusted rate	Calculated
Downstream Operator Labor Hours Per Year	3,990 labor-hrs/year	Operation-specific operator labor-hours per process operation – see SuperPro file for details<br>Model assumes 60% of labor hours devoted to process operations	Calculated
Downstream Labor Cost Per Year	$275,286/year	Based on Downstream Operator Basic Pay Rate of $30/hr and including 40% for benefits, 10% for operating supplies, 20% for supervision and 60% for administration = $69/hour adjusted rate	Calculated

2 Environmental Health and Safety

An environmental health and safety assessment was also conducted for this case study. Results are shown in Supplementary Tables 2 – 4 and interpretation is provided in the Results and Discussion sections of the manuscript.

Supplementary Table 2. Summary of mass indices (MI = Mass of Species per batch/Mass of Griffithsin per batch) for chemicals involved in upstream and downstream processes.

	Component	Input Mass per Batch (g)	Input Mass Index (g component/g Griffithsin API)	Output Mass per Batch (g)	Output Mass Index (g component/g Griffithsin API)
**Upstream**	Iron EDTA	21	0.104	11	0.051
	Calcium Nitrate (Tetrahydrate)	1448	6.88	713	3.39
	Magnesium Sulfate (Heptahydrate)	555	2.64	273	1.30
	Potassium Nitrate	716	3.40	353	1.67

	Component	Input Mass per Batch (g)	Input Mass Index (g component/g Griffithsin API)	Output Mass per Batch (g)	Output Mass Index (g component/g Griffithsin API)
	Manganese Sulfate (Monohydrate)	1.75	0.008	0.867	0.004
	Boric Acid	3.26	0.015	1.606	0.008
	Copper Sulfate (pentahydrate)	0.09	0.0004	0.044	0.0002
	Sodium Molybdate (Dihydrate)	0.029	0.0001	0.014	0.00007
	Zinc Sulfate (Dihydrate)	0.172	0.0008	0.085	0.0004
	Phosphoric Acid (30%)	373	1.77	183.6	0.872
**Downstream**	Biomass w/ TMV	578,050	2,746	578,050	2,746
	Sodium Acetate	5,128	24.3	5,128	24.3
	Sodium Chloride	12,513	59.4	12,350	58.7
	Ascorbic Acid	2,200	10.45	2,200	10.45
	Sodium Metabisulfite	890	4.22	890	4.22
	Tris HCl	32	0.15	32	0.15
	$MgCl_2$	12,070	57.3	12,070	57.3
	KCl	9	0.04	4.7	0.02
	NaH_2PO_4	50	0.24	28	0.13
	KH_2PO_4	12	0.057	6.3	0.03
	Bentonite	12,678	60.02	12,678	60.2
	NaOH	40,728	193	40,728	193
	H_3PO_4	52,950	252	52,950	252

Supplementary Table 3. Summary of classification of chemicals used in Griffithsin production for different environmental impact groups. A = highest impact, B = medium impact and C = low impact. In this analysis A is assigned a value of 1, B is assigned a value of 0.3 and C is assigned a value of 0.

	Component	Resources (Input)	Grey Input (Input)	Component Risk (Input + Output)	Organisms (Input + Output)	Air (Output)	Water/ Soil (Output)
**Upstream**	Iron EDTA	B	C	C	B	C	A
	Calcium Nitrate (Tetrahydrate)	C	C	C	B	C	A
	Magnesium Sulfate (Heptahydrate)	B	C	C	C	C	C
	Potassium Nitrate	C	C	C	B	C	A
	Manganese Sulfate (Monohydrate)	B	C	C	B	C	C
	Boric Acid	C	C	C	B	C	C
	Copper Sulfate (pentahydrate)	C	C	C	B	A	B
	Sodium Molybdate (Dihydrate)	C	C	C	B	C	C
	Zinc Sulfate (Dihydrate)	B	C	C	A	C	C
	Phosphoric Acid (30%)	C	C	C	A	A	A
**Downstream**	Biomass w TMV	N/A	N/A	C	B	C	A*
	Sodium Acetate	B	C	C	B	C	B
	Sodium Chloride	C	C	C	C	C	C
	Ascorbic Acid	C	C	C	C	C	B
	Sodium Metabisulfite	C	C	C	B	C	C
	Tris HCl	C	C	C	C	C	A
	$MgCl_2$	C	C	C	B	C	C
	KCl	C	C	C	C	C	C
	NaH_2PO_4	C	C	C	C	C	B
	KH_2PO_4	B	C	C	C	B	B

	Component	Resources (Input)	Grey Input (Input)	Component Risk (Input + Output)	Organisms (Input + Output)	Air (Output)	Water/ Soil (Output)
	Bentonite	C	C	C	B	C	C
	NaOH	C	C	B	A	C	C
	H_3PO_4	C	C	C	A	A	A

*Assumes that the TMV has not been chemically or thermally inactivated

Supplementary Table 4. Summary of environmental factors (EF) and environmental indices (EI = EF x MI) for chemicals involved in Griffithsin production. EF values will range from 0 (no environmental, health and/or safety impact) to 1 (high environmental, health and/or safety impact).

	Component	Input EF	Output EF	Input EI	Output EI
**Upstream**	Iron EDTA	0.15	0.325	0.016	0.017
	Calcium Nitrate (Tetrahydrate)	0.075	0.325	0.52	1.10
	Magnesium Sulfate (Heptahydrate)	0.075	0	0.20	0
	Potassium Nitrate	0.075	0.325	0.26	0.54
	Manganese Sulfate (Monohydrate)	0.15	0.075	0.001	0.0003
	Boric Acid	0.075	0.075	0.001	0.0006
	Copper Sulfate (pentahydrate)	0.075	0.04	0	0
	Sodium Molybdate (Dihydrate)	0.075	0.075	0	0
	Zinc Sulfate (Dihydrate)	0.25	0.075	0	0
	Phosphoric Acid (30%)	0.25	0.75	0.44	0.654
**Downstream**	Biomass w TMV	N/A	0.325	N/A	892
	Sodium Acetate	0.15	0.15	3.6	3.6
	Sodium Chloride	0	0	0	0
	Ascorbic Acid	0	0.075	0	0.78
	Sodium Metabisulfite	0.075	0.075	0.32	0.32
	Tris HCl	0	0.25	0	0.04
	$MgCl_2$	0.075	0.075	4.3	4.3

	Component	Input EF	Output EF	Input EI	Output EI
	KCl	0	0	0	0
	NaH_2PO_4	0	0.075	0	0.01
	KH_2PO_4	0.075	0.15	0.004	0.005
	Bentonite	0.075	0.075	4.5	4.5
	NaOH	0.325	0.325	63	63
	H_3PO_4	0.25	0.75	63	189
